# Scar quality examination comparing titanium-coated suture material and non-coated suture material on flap donor sites in reconstructive surgery

**DOI:** 10.1186/s12893-020-00932-3

**Published:** 2020-11-03

**Authors:** Laura K. Berninghausen, Georg Osterhoff, Stefan Langer, Lukas H. Kohler

**Affiliations:** Department of Orthopedic, Trauma and Plastic Surgery, Leipzig University Hospital, Liebigstraße 20, 04103 Leipzig, Saxony Germany

**Keywords:** Suture, Titanium coated, Wound healing, Scar quality, POSAS score

## Abstract

**Background:**

Wound healing and scar quality after trauma are subject to impairment through excessive wound healing, chronic wound or even surgical site infections. Optimizing the process of scar formation and skin healing is crucial in virtually all fields of medicine. In this regard, we tested the possible usage and advantages of titanium coated suture material.

**Methods:**

We performed a prospective observational cohort study including 30 patients who underwent soft tissue reconstruction. One half of the donor flap site was sutured with titanium coated suture material, while the other half was closed with non-coated sutures. Scar quality of the donor flap site was assessed by photographs and POSAS scores on days 2–5, 14, 42, 72 and 180 postoperatively.

**Results:**

No difference between the titanium coated sutures and non-coated sutures was seen in the POSAS assessment, neither for the patient scale at 14, 42, 72 and 180 days, nor for the observer scale on the same dates. Comorbidities like diabetes, chronic renal failure and smoking as well as the BMI of each patient affected the wound healing process to an equal degree on both sides of the suture.

**Conclusions:**

No difference between the titanium coated and non-titanium-coated suture material was seen in the POSAS assessment in regard to scar quality and wound healing. The titanium-coated suture material can be considered to be equally as effective and safe in all qualities as the non-titanium-coated suture material, even in patients with comorbidities.

*Clinical trial register* This study is registered at the German Clinical Trials Register (DRKS) under the registration number DRKS00021767. (https://www.drks.de/drks_web/navigate.do?navigationId=trial.HTML&TRIAL_ID=DRKS00021767)

## Background

As a result of trauma, either by accident or intent procedure, the skin is subject to a wound healing process resulting in the formation of a mature scar and therefore maintaining the integrity of the skin [1, 2].

Deviation from the physiological wound healing process, such as excessive wound healing, chronic wound or even surgical site infections, can impair the scar quality and the adequate physical function of the skin [2–4]. A surgical suture’s tissue integration and biocompatibility are decisive factors for ideal wound healing, therefore the optimal choice of suture material is still up for discourse [5, 6].

Surgical site infection hast been assessed to arise in 5% of all medical procedures in hospitals [7], resulting in pain and discomfort for the patients and prolonged hospitalization and additional costs to the health system [8]. Not only implanted foreign bodies, yet also suture material have to be taken into consideration as a host for biofilm and therefore a potential source of infection [7, 9, 10]. In order to surmount this issue, the coating of suture material not only with chemicals such as triclosan [11–16], yet also with metal legions is not uncommon in medicine. Silver nanoparticle-coatings on silk suture for example have proven to be successful in the prevention of surgical site infections [17–20].

Titanium as a metal legion is renowned for excellent bone to implant bonding [21, 22], high biocompatibility due to low allergic potential [23], resistance to corrosion [24] and limited complications like wound dehiscence, infection and pain [25–27]. Therefore, titanium is commonly used for medical products, whether it be as orthopedic implants [28], titanium clips in cardiology and neurology [29–31], auditory ossicle replacements [32], endoprosthetic surgery and osteosynthesis in dentistry [24, 33], nickel-titanium wire for closure in cleft lip procedures [25], titanium surgical tacks in gynecology [34] or as titanium coated meshes in abdominoplasty [35–38]. In the latter for example, it has shown to provoke less severe late inflammatory processes, greater tissue maturation and collagen disposition in comparison to a non-titanium-coated polypropylene mesh [37].

These qualities of titanium in medical products raise the question whether titanium, if used as a coating for suture material, could also be used to improve would healing and scar quality.

Wound healing is an immensely difficult and interference-prone process which needs to be assisted at its best and optimized constantly, especially in patients with comorbidities that could compromise ideal healing and scar formation [1–3].

In this study, we therefore aimed to further evaluate the possible usage and advantages of titanium coated suture material with regard to wound healing, surgical site infections and scar quality in reconstructive and plastic surgery. This was exemplified on flap surgery donor sites.

## Methods

### Patient collective

We performed a prospective observational cohort study. The materials of this study have been drafted from patients aged 18 and older who had given their informed consent. All patients received soft tissue reconstruction via free flap surgery at the University Hospital Leipzig from August 2018 to October 2019. Flaps included ALT-, latissimus dorsi-, DIEAP-, parascapular- and gastrocnemius flaps. In total, 30 patients (7 females, 23 males) with a median age of 60 (26 to 92) were included. Baseline data comprised of gender, BMI, comorbidities, duration of hospitalization after surgical intervention and wound healing with Patient and Observer Scar Assessment Score/POSAS (Additional files 1, 2).

After raising the flap, the donor site wound was sutured continuously and intracutaneously with titanium coated suture material (Seratan^®^ 2-0, titanium coated [Serag Wiessner GmbH & Co. KG., Naila, Germany]) on one half and non-titanium-coated suture material (Seralon^®^ 2-0, non-titanium-coated [Serag Wiessner GmbH & Co. KG., Naila, Germany]) on the other (Additional file 3). The study was performed in a single blinded design with patients not knowing which half was sutured with titanium coated or non-titanium-coated material. Seratan^®^ is priced at 264.94€ per unit (24 pieces) including tax and shipping, Seralon^®^ at 168.20€ per unit (24 pieces).

### *Scar assessment *via* POSAS scores and clinical photography*

On days 2–5 post-surgery, wound visits were performed. On day 10, the stitches were removed and on day 14, POSAS (Patient and Observer Scar Assessment Scale) scores for both sides of the suture were executed by the patient and an observer. Wound visits and POSAS scores were again carried out on day 42, 72 and 180. Photographical documentation was performed continuously during inpatient and outpatient follow up dates (Additional files 3, 4). The POSAS consists of both a Patient Scale and an Observer Scale. Both scales contain six items that are scored numerically on a ten-step scale with 10 indicating the worst imaginable scar or sensation and 1 corresponding to the situation of normal skin. Together they make up the total score of the scale.

### Statistical analysis

Post-test analysis was done using SPSS for Windows V24.0 (IBM, Chicago, IL, USA). All data is reported as frequencies with percentages (%) or mean and standard deviation (SD) with ranges.

A paired t-test was used to detect differences in means between Seratan^®^ and Seralon^®^ for continuous data. The level of significance was defined as p < 0.05.

## Results

In total, 30 patients with a mean age of 60 years (SD 16, range, 26 to 92; 7 females, 23 males) were included into the final analysis. The most frequent flap entity was the anterior lateral thigh flap (n = 23), followed by latissimus dorsi flaps (n = 3), deep inferior epigastric artery perforator flaps (n = 2), gastrocnemius flaps (n = 1), and parascapular flaps (n = 1). The mean hospital stay after surgery was 13 days (SD 7, range 5 to 37).

The patients’ mean Body Mass Index was 27.0 kg/m^2^ (SD 5.9, range 18 to 44). Preexisting comorbidities that are known to affect wound healing were diabetes in 15 patients (50%), smoking in 13 (43%) and chronic renal failure in 10 patients (33%). No patient received immunosuppressive medication.

All patients’ donor site wounds were closed with above named suture material in a 50:50 fashion. The mean wound length per patient sutured with titanium-coated material was 11.2 cm (SD 2.6) and 10.9 cm (SD 2.1) for non-coated sutures (p = 0.293). Complications occurred in four patients. This included one suture fistula (Seratan^®^), one superinfected hematoma (Seralon^®^), one superficial wound necrosis (Seralon^®^ + Seratan^®^), and one hematoma which had to be revised (Seratan^®^). All other complications were managed in a conservative fashion.

**Fig. 1:**
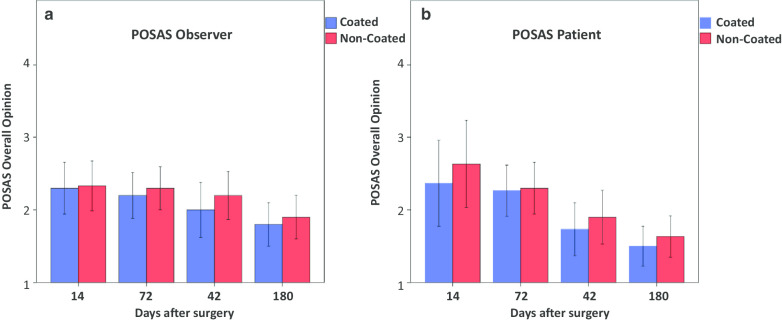
POSAS overall opinion of observers (**a**) and patients (**b**) on follow-up days 14, 42, 72 and 180 for titanium-coated suture material (blue bar) and non-coated suture material (red bar)

**Fig. 2:**
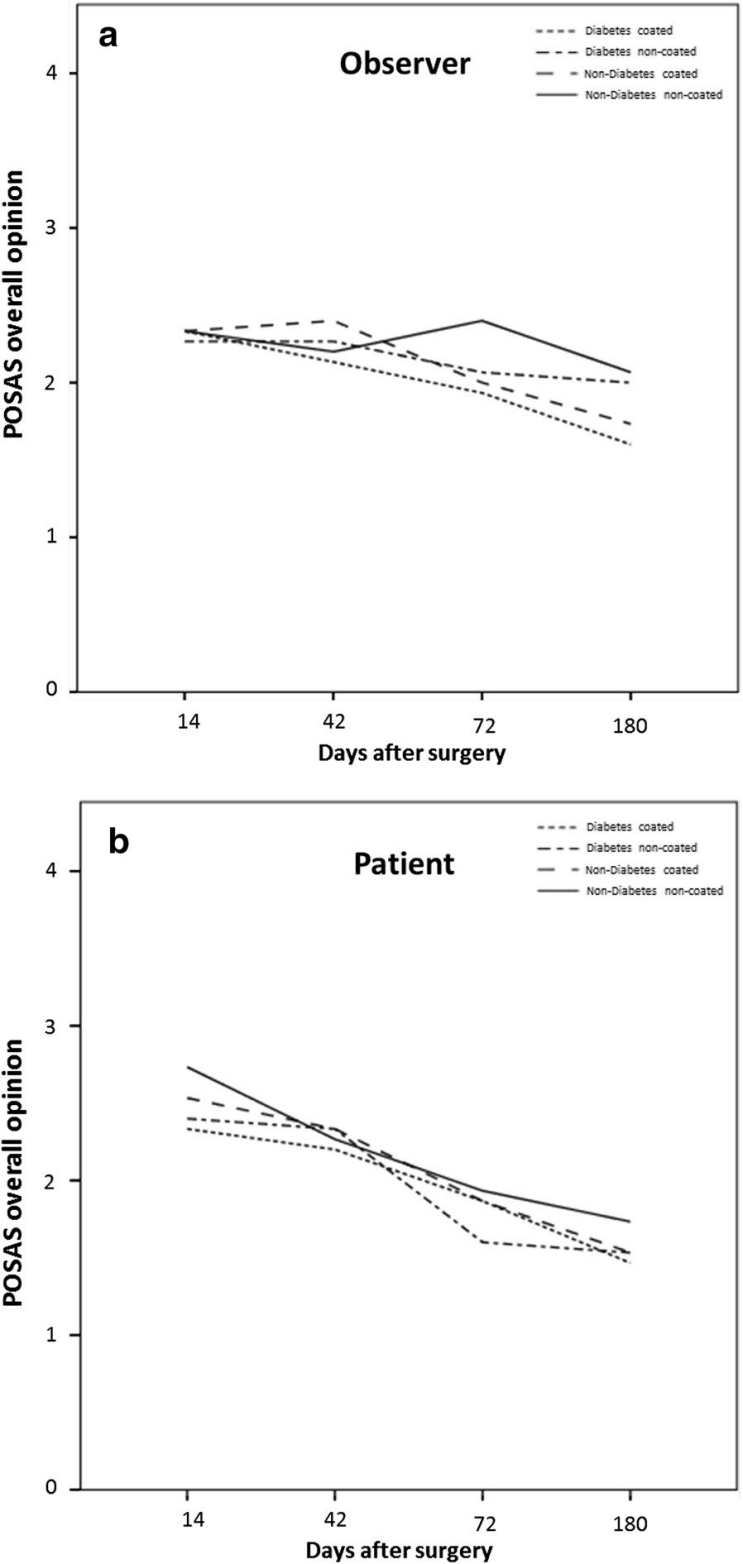
Correlation analysis between POSAS overall opinion for observers (**a**) and patients (**b**) and diabetes mellitus type II on follow-up days 14, 42, 72 and 180 for titanium-coated suture material and non-coated suture material

**Fig. 3:**
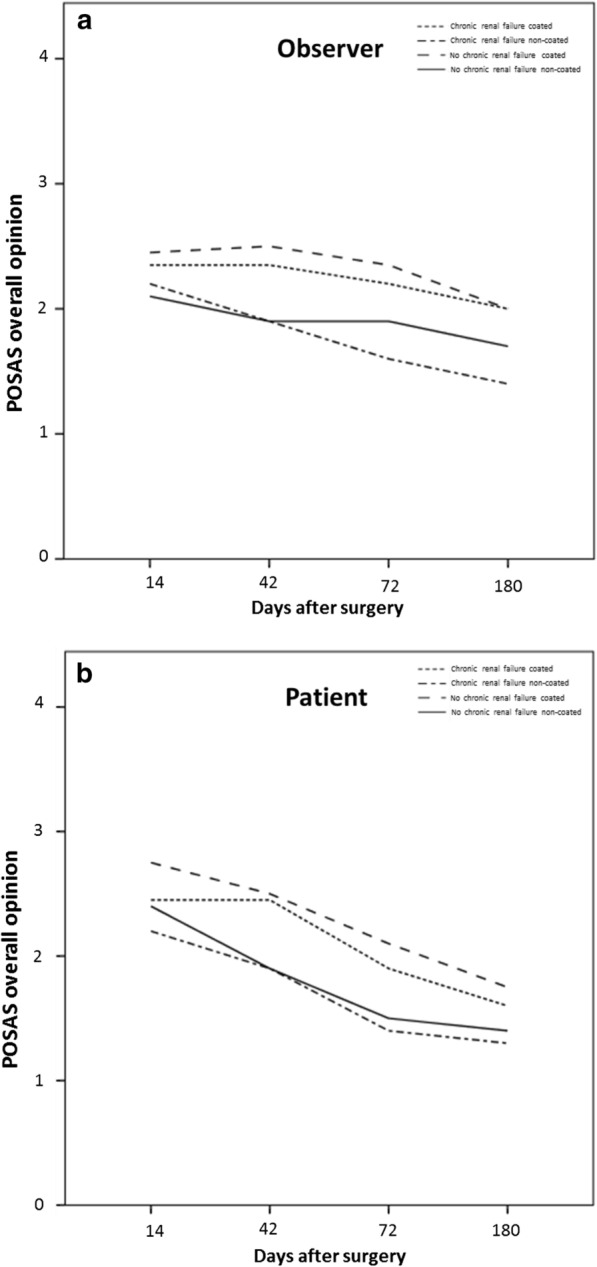
Correlation analysis between POSAS overall opinion for observers (**a**) and patients (**b**) and chronic renal failure on follow-up days 14, 42, 72 and 180 for titanium-coated suture material and non-coated suture material

**Fig. 4:**
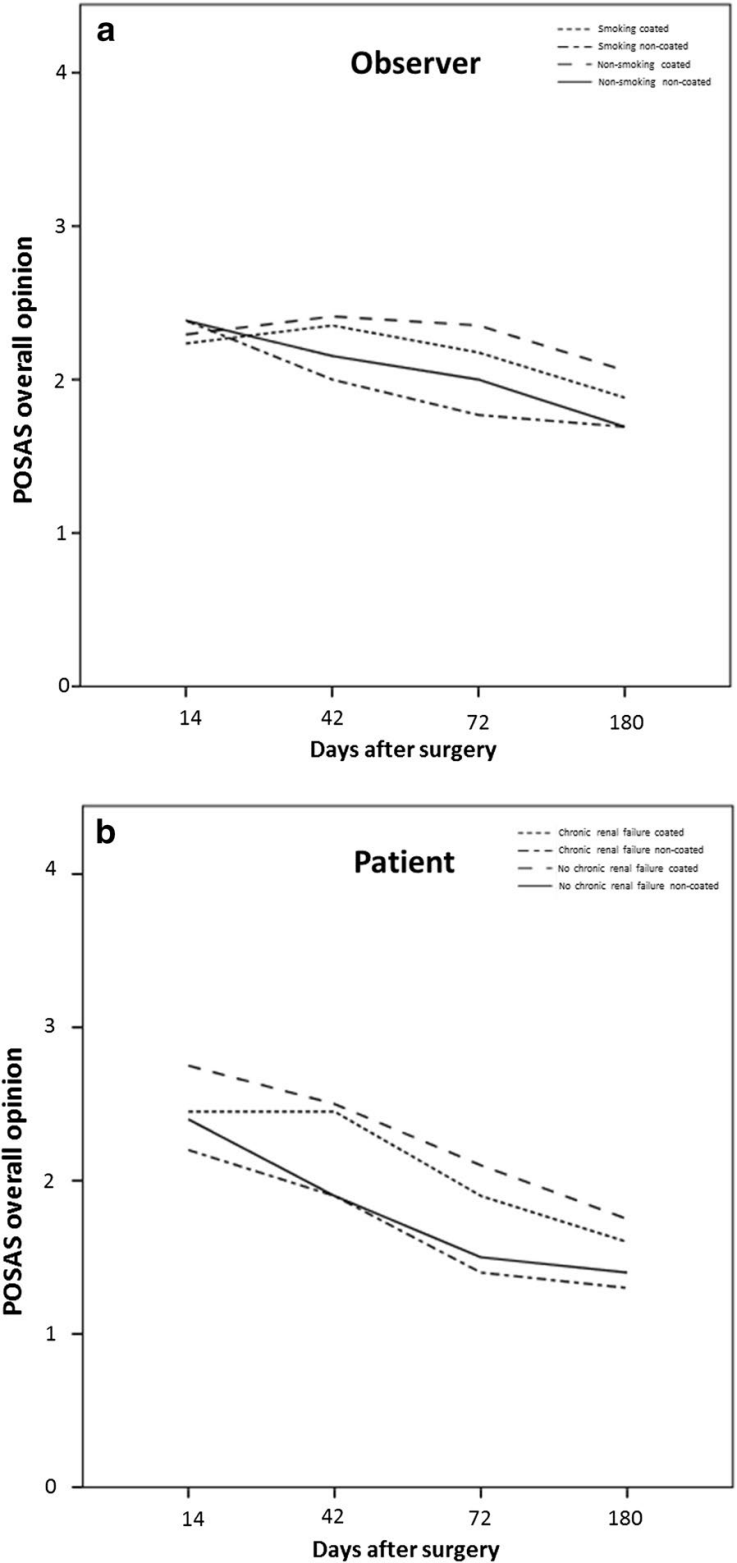
Correlation analysis between POSAS overall opinion for observers (**a**) and patients (**b**) and smoking on follow-up days 14, 42, 72 and 180 for titanium-coated suture material and non-coated suture material

**Fig. 5:**
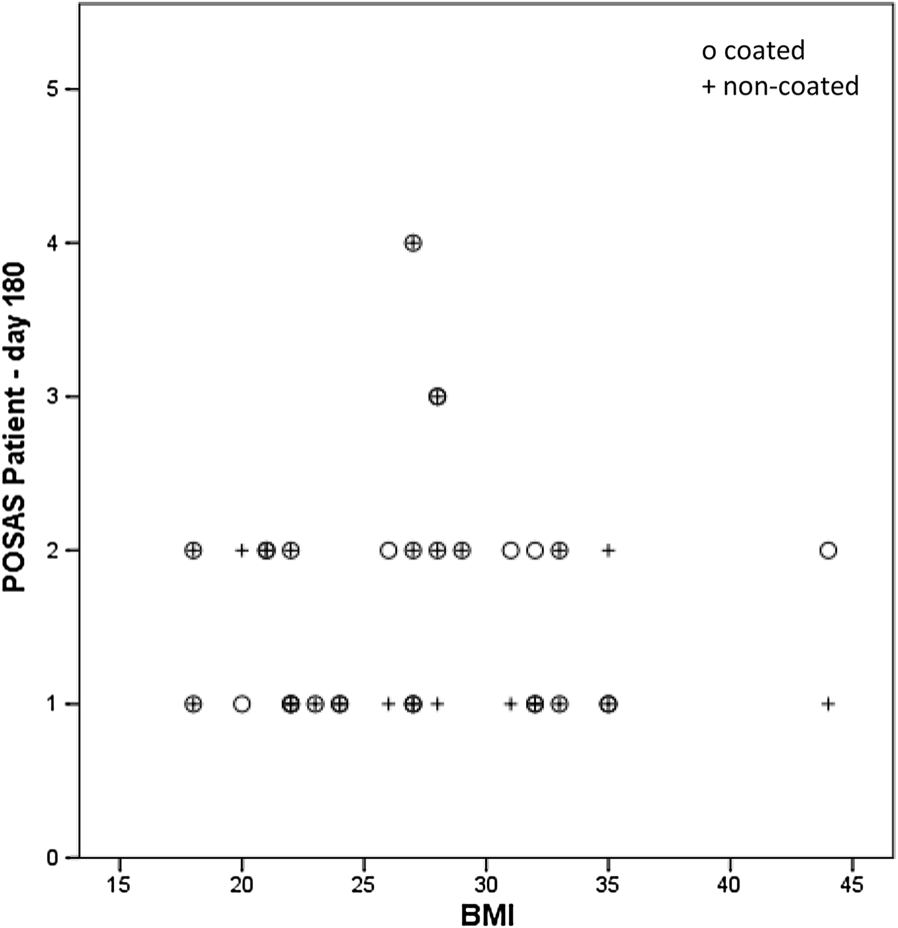
Correlation analysis between POSAS overall opinion and Body Mass Index (BMI) on follow-up day 180 for titanium-coated suture material and non-coated suture material

No significant difference between the titanium coated and non-titanium coated sutures was seen in the POSAS assessment, neither for the patient scale at 14 days (p = 0.161), at 42 days (p = 0.787), at 72 days (p = 0.433) or at 180 days (p = 0.293), nor for the observer scale at 14 days (p = 0.787), at 42 days (p = 0.522), at 72 days (p = 0.184) or at 180 days (p = 0.375). However, there is a tendency for a slightly better overall opinion in all follow-ups for the titanium coated material in both groups, patients and observers (Fig. 1). When taking into consideration comorbidities like diabetes (Seratan^®^: p = 0.808, Seralon^®^: p = 0.484), chronic renal failure (Seratan^®^: p = 0.297, Seralon^®^: p = 0.244) or smoking (Seratan^®^: p = 0.459, Seralon^®^: p = 0.562) while comparing the POSAS scores on day 180, no significant difference between the two suture materials could be detected (Figs. 2, 3 and 4). Furthermore, comparing overall patient satisfaction on day 180 on coated and non-coated wound closure sites in correlation to the BMI of each patient did not show any significant difference in scar quality (Seratan^®^: p = 0.541, Seralon^®^: p = 0.647, Fig. 5).

## Discussion

Wound healing and scar quality are an important part of virtually every field of medicine. The choice of suture material, as an immense factor of impact for the scar formation and healing abilities, is of great importance when it comes to optimizing these processes. This is valid especially in patients with comorbidities which compromise ideal healing and scar formation, like diabetes mellitus, chronic renal failure or the indulgence in nicotine [1–3]. Our findings indicate that titanium coated suture material can be seen as equivalently effective and safe as non-titanium-coated suture material.

In this pilot study with 30 patients, scar quality was assessed on flap surgery donor sites with the help of POSAS score evaluation and photography, while comparing titanium coated sutures with non-titanium-coated sutures. The coating of medical material with titanium material can be seen as leadoff technique in medicine, therefore scientific literature on this topic is rare. Notwithstanding, our findings can be seen as consistent with the advantages of coating suture material with chemicals [11–16] or metal legions, like the prevention of surgical site infections [17–20]. They were also in accordance with beneficial qualities of titanium in medical products, like excellent bone to implant bonding, high biocompatibility due to low allergic potential, resistance to corrosion and limited complications like wound dehiscence, infection and pain [21–27].

The titanium coated suture material was equivalent to the non-coated-suture material in regard to scar quality and wound healing. Even the presence of comorbidities did not have any impact on these qualities. However, it should be emphasized that high satisfaction in regard to scar quality was achieved in both groups which also increased over the course of time. Additionally, titanium coated sutures presented slightly better results in regard to wound healing than non-titanium-coated sutures. This data suggests the possibility of using titanium coated material in surgical procedures for wound closure in the future. Our findings support the results of Saalabian et al. in which titanium coated suture material showed significantly lower signs of inflammation in small wounds of the hand and forearm [39].

The limitations of this study include its small sample size and the limited variability in surgical sites. A more extensive clinical study and a cost efficiency analysis would have to be planned to not only show clinical evidence, but also practicability in the long term. Nevertheless, titanium coated suture material can be considered as equally effective and safe as non-titanium-coated suture material in regard to wound healing, scar quality and surgical site infection. Combining the advantages of titanium legions in medical products with benefits of coating suture material in order to minimize surgical site infections and therefore receiving optimum wound healing and scar formation was the intention behind the development of the suture material and this study. However, our research suggests that the production of the titanium coated suture material will not be able to deliver superior results to the non-titanium-coated suture material. Considering the higher price as mentioned above, we currently see no reason for a standardized wound closure with titanium coated sutures in our patients.

## Conclusions

Titanium coated suture material brings forward as equally adequate results in scar quality and wound healing in flap surgery donor sites as non-titanium-coated suture material. The coating of medical products in general with titanium seems to have a positive impact on wound healing and provides decreased complications like wound dehiscence and surgical site infection [25–27]. Nevertheless, the utilization of titanium coated suture material on flap surgery donor sites, even in patients with comorbidities or immunocompromization, will not be superior to non-titanium-coated suture material.

## Supplementary information

**Additional file 1.** Patient Observer and Patient Scale Assessment Scale for Observers. The scale is designed for scar evaluating of professionals and contains six items that are scored numerically on a ten-step scale. The items should be compared to regular skin at a comparable anatomic area. To ensure quality of examination, more than one professional should evaluate the POSAS. The items for professionals include Vascularity, Pigmentation, Thickness, Relief, Pliability and Surface Area. Furthermore, it asks for an Overall Opinion. With kind permission of P.P.M. van Zuijlen, Beverwijk-NL.

**Additional file 2.** Patient Observer and Patient Scale Assessment Scale for patients. The scale is designed for scar evaluating of non-professionals/patients and contains six items that are scored numerically on a ten-step scale. Items include pain, itching, color difference, stiffness, thickness and irregularity in comparison with regular skin. Also, just like in the Observers Score, it asks for an Overall Opinion. With kind permission of P.P.M. van Zuijlen, Beverwijk-NL.

**Additional file 3.** Intraoperative photographic documentation of wound closure in ALT donor site. You can see the 50:50 fashion that was assessed using titanium coated suture material (Seratan^®^) for one half of the wound closure and non-titanium coated material (Seralon^®^) for the other half. Markings were only made for illustration reasons and removed immediately after taking the photograph to ensure single blinding of the patient.

**Additional file 4.** Photographic documentation of wound closure in ALT donor site on day 14.

## Data Availability

All data is contained within the manuscript. The datasets used and analyzed during the current study available from the corresponding author on reasonable request.

## Abbreviations

POSAS: Patient and Observer Scar Assessment Scale
et al.: Et alii. and others
GmbH and Co. Kg.: Gesellschaft mit beschränkter Haftung & Compagnie KG (GmbH & Co. KG) is a limited partnership with, typically, the sole general partner being a limited liability company
