# Supplementary figures and images for: Scar quality examination comparing titanium-coated suture material and non-coated suture material on flap donor sites in reconstructive surgery
Source: BMC Surg. 2020 Nov 3;20:268. doi: 10.1186/s12893-020-00932-3 (PMC7640681; doi:10.1186/s12893-020-00932-3)

Additional file 1


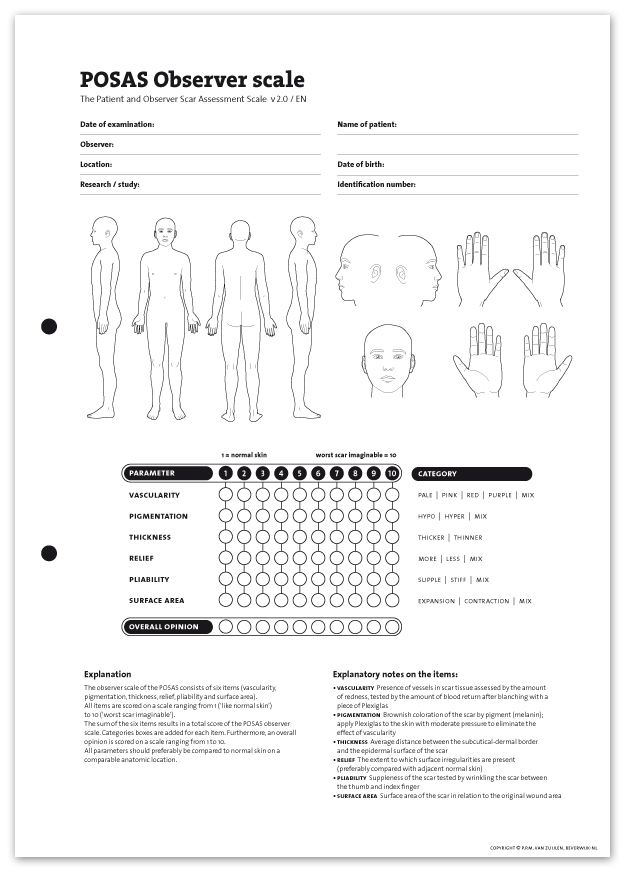

Supplement: Supplementary file 1 — Additional file 1. Patient Observer and Patient Scale Assessment Scale for Observers. The scale is designed for scar evaluating of professionals and contains six items that are scored numerically on a ten-step scale. The items should be compared to regular skin at a comparable anatomic area. To ensure quality of examination, more than one professional should evaluate the POSAS. The items for professionals include Vascularity, Pigmentation, Thickness, Relief, Pliability and Surface Area. Furthermore, it asks for an Overall Opinion. With kind permission of P.P.M. van Zuijlen, Beverwijk-NL. [file 12893_2020_932_MOESM1_ESM.docx]

Additional file 2


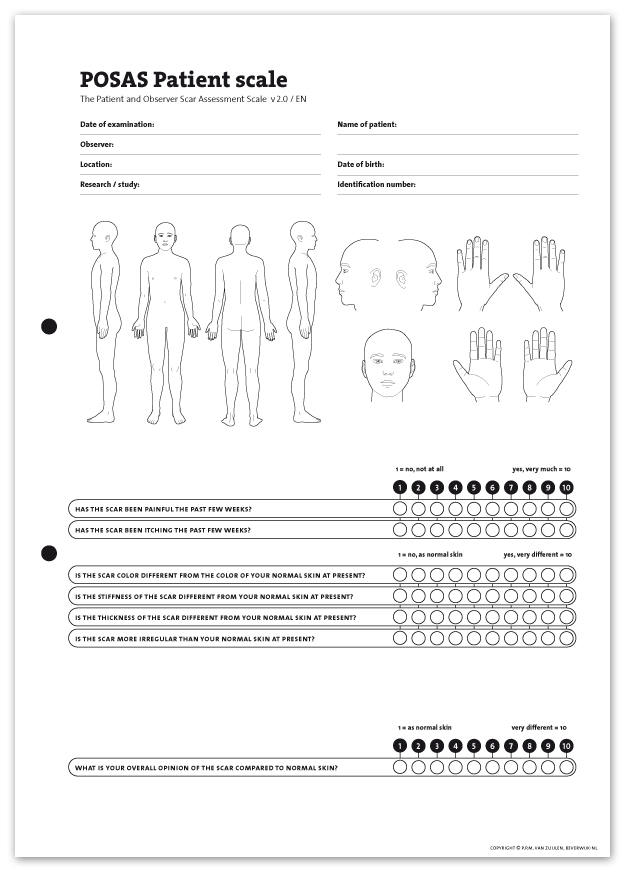

Supplement: Supplementary file 2 — Additional file 2. Patient Observer and Patient Scale Assessment Scale for patients. The scale is designed for scar evaluating of non-professionals/patients and contains six items that are scored numerically on a ten-step scale. Items include pain, itching, color difference, stiffness, thickness and irregularity in comparison with regular skin. Also, just like in the Observers Score, it asks for an Overall Opinion. With kind permission of P.P.M. van Zuijlen, Beverwijk-NL. [file 12893_2020_932_MOESM2_ESM.docx]

Additional file 3


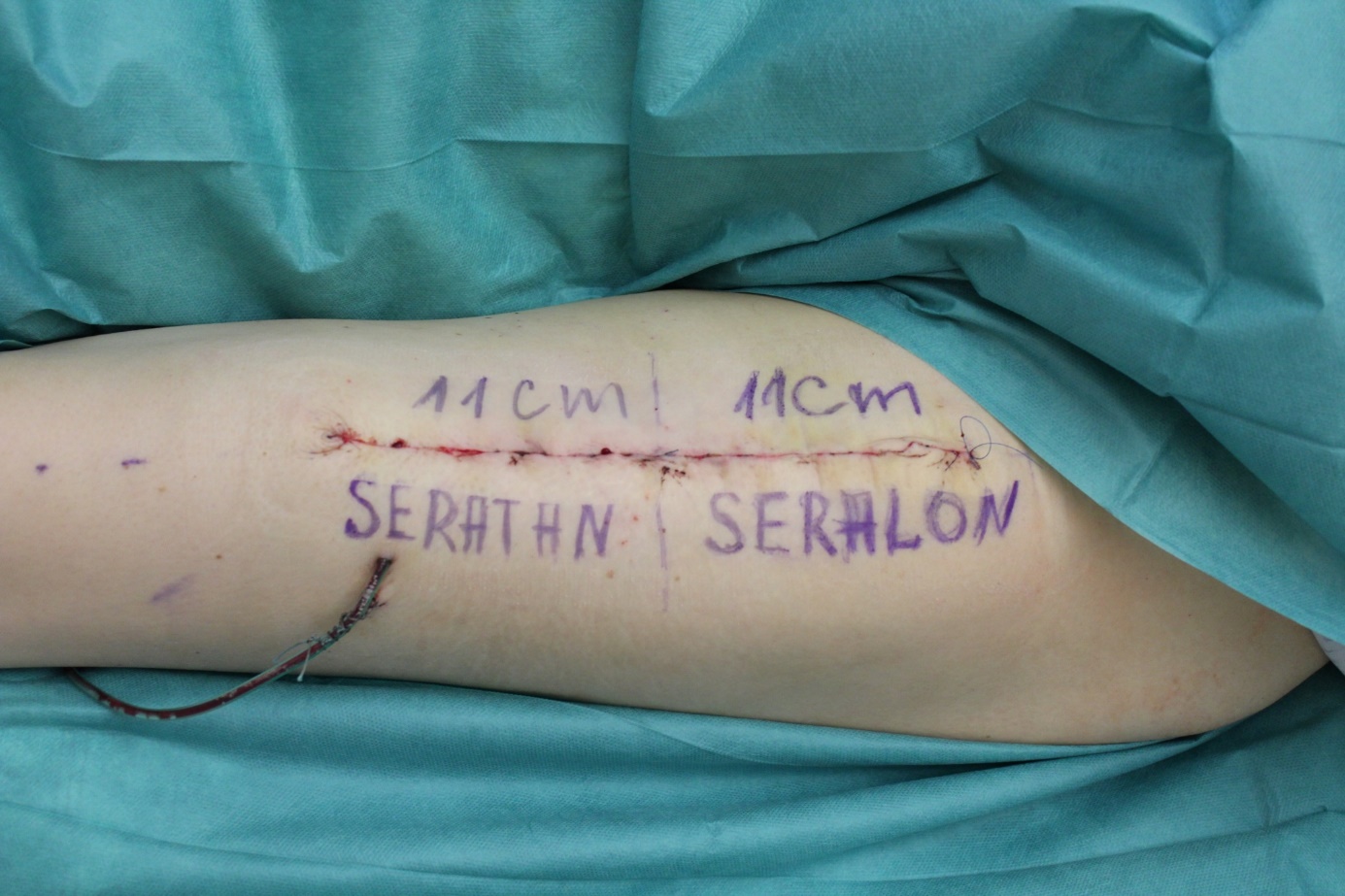

Supplement: Supplementary file 3 — Additional file 3. Intraoperative photographic documentation of wound closure in ALT donor site. You can see the 50:50 fashion that was assessed using titanium coated suture material (Seratan®) for one half of the wound closure and non-titanium coated material (Seralon®) for the other half. Markings were only made for illustration reasons and removed immediately after taking the photograph to ensure single blinding of the patient. [file 12893_2020_932_MOESM3_ESM.docx]

Additional file 4


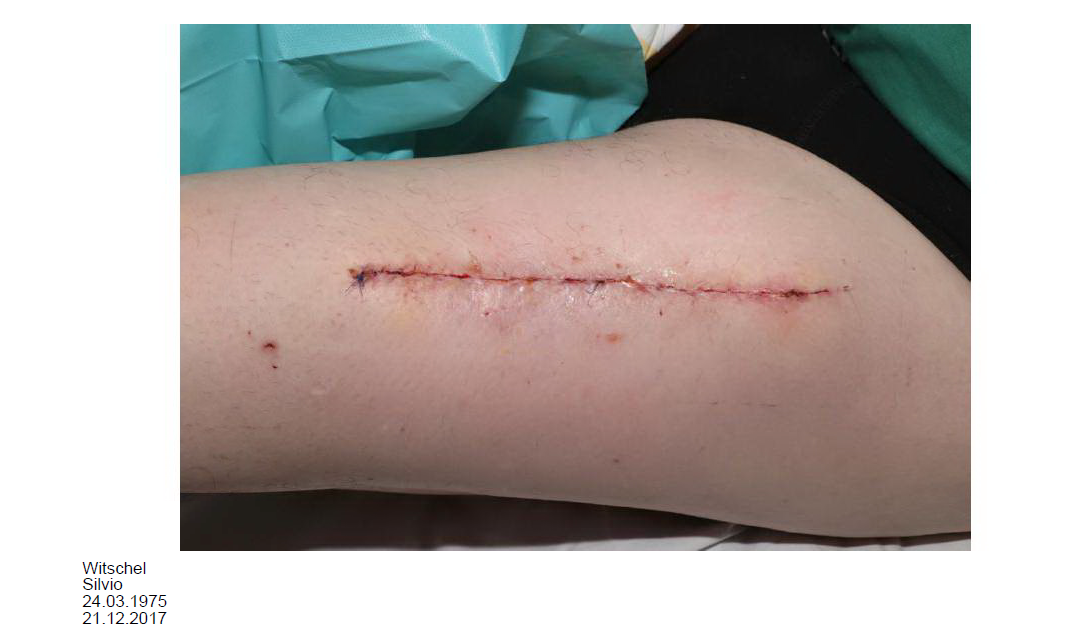

Supplement: Supplementary file 4 — Additional file 4. Photographic documentation of wound closure in ALT donor site on day 14. [file 12893_2020_932_MOESM4_ESM.docx]
